# Une cause rare des tumeurs du médiastin postérieur: le kyste hydatique médiastinal

**DOI:** 10.11604/pamj.2016.25.122.9949

**Published:** 2016-10-27

**Authors:** Hicham Souhi, Adil Zegmout, Hicham Janah, Hanane El ouazzani, Ismail Abderrahmani Rhorfi, Ahmed Abid

**Affiliations:** 1 Service de Pneumologie, Hôpital Militaire d’Instruction Mohammed V, Rabat, Maroc

**Keywords:** Kyste hydatique, tumeur, médiastin, Hydatid cyst, tumor, mediastinum

## Abstract

Le kyste hydatique médiastinal est extrêmement rare même en pays d’endémie, représentant 0 à 4% de toutes les localisations hydatiques. Nous rapportons l’observation d’un patient âgé de 50 ans, admis au service pour une masse para-vertébrale dorsale gauche; avec à la radiographie thoracique une opacité postérieure à projection basale gauche. La TDM thoracique montre une masse médiastinale postérieure de la gouttière costo-vetébrale gauche étendue en regard de D9 jusqu’à D11. L’IRM confirme l’existence de la masse médiastinale postérieure avec extension endocanalaire et refoulement médullaire, faisant évoquer en 1^er^ un schwanome kystique. Devant ces données radiocliniques, l’origine néoplasique est évoquée et une ponction biopsie transpariétale réalisé a ramené un prélèvement paucicellulaire fait d’un matériel éosinophile translucide avec aspect en coup de peine compatible avec un KH. La sérologie hydatique est positive. Le diagnostic de KH est retenu et le patient a bénéficié d’une thoracotomie qui a révélé un KH médiastinal; confirmé par l’étude anatomopathologique. Les suites opératoires étaient simples. La localisation médiastinale du KH est très rare et pose un problème de diagnostic différentiel avec les autres tumeurs médiastinales.

## Abstract (en)

Mediastinal hydatid cyst is extremely rare even in endemic areas, representing 0-4% of all hydatid cyst locations. We report the case of a 50-year old patient admitted to our Department with a mass in the left dorsal paraspinal region; chest X-ray showed posterior left basal opacity. Chest CT scan showed posterior mediastinal mass located in the left costovertebral gutter extending from D9 to D11. MRI confirmed the existence of a posterior mediastinal mass with endocanalar extension and spinal cord compression, first evoking cystic schwannoma. These radioclinical data were consistent with a neoplastic origin; a transparietal biopsy was performed which showed a paucicellular specimen composed of translucent eosinophilic material with appearance just barely compatible with hydatid cyst. Hydatic serology was positive. The diagnosis of hydatid cyst was retained and the patient underwent thoracotomy which revealed mediastinal hydatid cyst, confirmed by histologic examination. The postoperative course was uneventful. Mediastinal location of hydatid cyst is very rare and poses a problem in differential diagnosis with other mediastinal tumors.

## Introduction

L’hydatidose est une parasitose causée par le développement chez l’homme de la forme larvaire du taenia Echinococcus. La localisation médiastinale du kyste hydatique est extrêmement rare représentant 0 à 4% de toutes les localisations hydatiques. Cette localisation pose volontiers un délicat problème diagnostique avec les lésions kystiques du médiastin.

## Patient et observation

**Figure 1:**
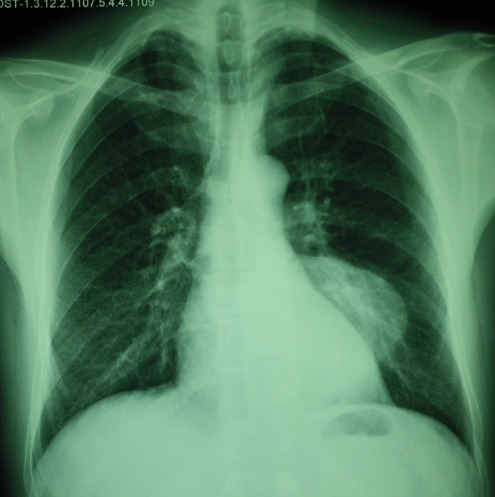
Radiographie thoracique montrant une opacité postérieure à projection basale gauche

**Figure 2:**
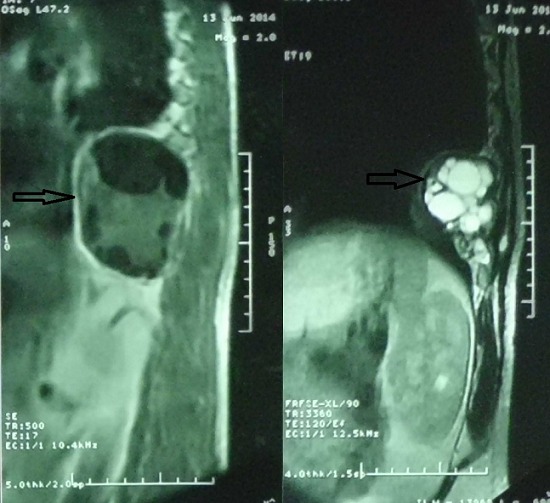
IRM médullaire objectivant l’existence d’une masse médiastinale postérieure de contours nets et irréguliers et de densité hétérogène tissulaire et kystique multi cloisonné avec extension endocanalaire et refoulement médullaire

Il s’agissait d’un homme de 50 ans, tabagique chronique à raison de 15 PA, sans autres ATCDS pathologiques particuliers et sans notion de contact avec les chiens, qui consultait pour une tuméfaction para vertébrale dorsale gauche évoluant depuis 3 ans. L’examen trouvait une masse para vertébrale dorsale gauche ferme faisant 5 cm de grand axe fixée au plan profond la peau en regard était saine. La radiographie du thorax a montré une opacité postérieure à projection basale gauche (Figure 1). L’échographie des parties molles était en faveur d’une masse pariétale dorsale gauche multivésiculaire avec extension endothoracique. La Tomodensitométrie thoracique avait mis en évidence: une masse médiastinale postérieure de la gouttière costo-vertébrale gauche en basithoracique étendue en regard de D9 jusqu’à D11 de contours nets et irréguliers et de densité hétérogène tissulaire et kystique multi cloisonné avec une calcification linéaire périphérique avec extension endocanalaire évoquant en 1^er^ une tumeur neurogène (type schwanome kystique). Le bilan était complété par une IRM médullaire qui avait confirmé l’existence de la masse médiastinale postérieure avec extension endocanalaire et refoulement médullaire, faisant évoquer en premier un schwannome kystique (Figure 2). Devant ces données radio cliniques; l’origine néoplasique était évoquée; une ponction biopsie transpariétale avait montré un prélèvement paucicellulaire fait essentiellement d’un matériel éosinophile translucide montrant un aspect en coup du peine compatible avec un kyste hydatique. La sérologie hydatique par la technique ELISA était positive. Le patient a alors bénéficié d’une thoracotomie qui révélait un kyste hydatique médiastinal confirmé par l’étude anatomopathologique. Les suites opératoires étaient simples sur un recul de 18 mois.

## Discussion

La localisation médiastinale de l’hydatidose est très rare même en zone d’endémie; elle varie entre 0,1 et 4% [1, 2]. La pathogénie de la localisation médiastinale du KH reste mal élucidée. Deux mécanismes peuvent être à l’origine de cette localisation: le parasite après avoir franchi le filtre hépatique et pulmonaire pénètre dans la circulation sanguine systémique et se fixe dans le médiastin; le second mécanisme est celui d’un cheminement par les voies chylifères et lymphatiques [1, 3–5]. Toutes les localisations dans le médiastin sont possibles avec une prédilection pour le médiastin postérieur [1, 3]. La symptomatologie clinique est non spécifique dominée par des signes de compression médiastinale le kyste hydatique du médiastin postérieur peut entraîner des douleurs et des signes neurologiques avec possibilité d’érosion des côtes et des vertèbres [1, 3, 6]. L’imagerie joue un rôle essentiel dans le diagnostic positif et le bilan d’extension des lésions. La radiographie standard montre un élargissement du médiastin associé ou non à une cardiomégalie. Les calcifications de la paroi sont rares mais représentent un argument diagnostique non négligeable et s’observent au cours des KH primitif du médiastin [1, 3, 5, 6]. L’échographie est un examen très performant qui permet de reconnaître le caractère liquidien et la paroi fine du kyste. L’image d’un « grelot » solide au sein du liquide; d’un décollement membranaire ou de vésicules filles; est fortement évocatrice du diagnostic mais rarement observée [1, 3, 5]. La TDM montre une masse de densité liquidienne bien limitée non modifiée par le produit de contraste; et dont les limites sont nettes [1, 3, 5, 7, 8]. Dans le cas de notre patient il s’agissait d’une masse de densité hétérogène tissulaire et kystique multi cloisonné. L’IRM est très peu utilisée dans cette localisation, elle permet de mieux préciser la topographie des ces lésions et les rapports avec les organes de voisinages. L’IRM peut aussi être utile au diagnostic en montrant les vésicules filles à l’intérieur du kyste [1, 3, 5–7, 9]. Le traitement est toujours chirurgical [3, 4, 9]. Le résultat est favorable dans la plupart des séries rapportées.

## Conclusion

La localisation médiastinale du KH est rare. L’imagerie doit normalement permettre le diagnostic positif; ainsi que le bilan d’extension aux organes de voisinages. Son traitement est chirurgical et l’évolution post-thérapeutique est souvent favorable.
